# Genetic, Clinical, and Sociodemographic Profile of Individuals with Diagnosis or Family History of Hypertrophic Cardiomyopathy: Insights from a Prospective Cohort

**DOI:** 10.3390/genes16091100

**Published:** 2025-09-17

**Authors:** Emerson de Santana Santos, Gabriel da Costa Kuhn, Antônio Guilherme Cunha de Almeida, João Victor Andrade Pimentel, Newton Vital Figueiredo Neto, Larissa Rebeca da Silva Tavares, Bárbara Letícia Lima dos Santos, Ana Beatriz Leite Aragão, Beatriz Carolina de Araujo Pereira, Caio da Silva Ferreira, Willian Moreira Leão e Silva, Cardiogenetics Research Group of Sergipe, Enaldo Vieira de Melo, Irlaneide da Silva Tavares, Antônio Carlos Sobral Sousa, Joselina Luzia Menezes Oliveira

**Affiliations:** 1 Department of Medicine, Federal University of Sergipe (UFS), Aracaju 49100-000, Braziljvapimentel@academico.ufs.br (J.V.A.P.); caioda_silvaferreira@outlook.com (C.d.S.F.); wmoreira200@gmail.com (W.M.L.e.S.); evmelo@academico.ufs.br (E.V.d.M.); acssousa@terra.com.br (A.C.S.S.); joselinamenezes@gmail.com (J.L.M.O.); 2 Postgraduate Program in Health Sciences, Federal University of Sergipe (UFS), Aracaju 49060-676, Brazil; 3 University Hospital, Federal University of Sergipe, Aracaju 49100-000, Brazil; irlaneidetavares@gmail.com; 4 University Hospital of Lagarto, Federal University of Sergipe, Aracaju 49100-000, Brazil; 5 Department of Medicine, Tiradentes University, Aracaju 49032-490, Brazil; gabriel.costa02@souunit.com.br (G.d.C.K.); newton.vital@souunit.com.br (N.V.F.N.); larissarebeca.tavares@gmail.com (L.R.d.S.T.); 6 Department of Fisheries and Aquaculture Engineering, Federal University of Sergipe (UFS), Aracaju 49100-000, Brazil; 7 Clinic and Hospital São Lucas/Rede D’Or São Luiz, Aracaju 49060-676, Brazil; 8 Division of Cardiology, University Hospital, Federal University of Sergipe (UFS), Aracaju 49100-000, Brazil

**Keywords:** hypertrophic cardiomyopathy, phenocopies, genetic testing, family history, cardiac amyloidosis

## Abstract

**Background**: Hypertrophic cardiomyopathy (HCM) is a genetic cardiac disorder characterized by left ventricular hypertrophy in the absence of secondary causes. This study aimed to investigate the genetic, clinical, and epidemiological profile of individuals with clinical HCM or a family history of sudden cardiac death (SCD). **Methods**: A total of 200 participants (58% male, median age 52 years) underwent genetic testing using a 19-gene panel associated with HCM and phenocopies. Variants were classified as pathogenic/likely pathogenic (P/LP), variants of uncertain significance (VUS), or negative. Clinical and imaging data were correlated with genetic findings. **Results**: P/LP variants were identified in 31% of individuals, while 40.5% carried VUS, and 28.5% tested negative. A positive genotype was more frequent among patients with clinical HCM (37.7%) than among those with only a family history (18.6%, *p* = 0.006). Sarcomeric mutations represented 77.4% of positive results, while 22.6% involved phenocopy genes, notably TTR (amyloidosis). Positive genotypes were significantly associated with a family history of SCD (68% vs. 46%, *p* = 0.004) and with greater interventricular septal thickness (17 mm vs. 15 mm, *p* < 0.001). **Conclusions**: Septal thickness >17 mm and family history of SCD were strong predictors of positive genetic results. These findings emphasize the importance of genetic screening and counseling in high-risk individuals and highlight the value of integrating genetic testing into clinical practice for diagnosis, risk stratification, and family management.

## 1. Introduction

Hypertrophic cardiomyopathy (HCM) is a relatively common hereditary cardiac condition, with an estimated prevalence of 1 in 500 individuals (0.2%) in the general population. It exhibits significant phenotypic and genetic variability, as well as a heterogeneous clinical course, affecting both sexes and diverse ethnic, cultural, and racial groups. Factors such as consanguinity and genetic diversity influence its prevalence and clinical expression, particularly in under-represented populations [1,2].

Building on this definition, the 2024 AHA/ACC/AMSSM/HRS/PACES/SCMR guidelines define HCM as left ventricular hypertrophy (LVH) not attributable to cardiac, systemic, or metabolic diseases. This encompasses cases with identified sarcomeric variants as well as those of indeterminate genetic etiology [3]. Histologically, HCM is marked by myocyte hypertrophy, disarray, and interstitial fibrosis, leading to diastolic dysfunction, ventricular arrhythmias, and an increased risk of sudden cardiac death (SCD), particularly in younger individuals. Diagnosis is primarily based on echocardiography or cardiac magnetic resonance imaging (MRI), the latter being the gold standard for detailed assessment. A wall thicknesses of 13–14 mm may also support diagnosis in individuals with a family history or a positive genetic test [3,4,5].

From a genetic perspective, advances since the 1990s have identified HCM’s monogenic inheritance pattern. Approximately 70% of cases result from mutations in sarcomeric genes such as MYBPC3 and MYH7, while other sarcomeric genes (e.g., TNNI3, TNNT2, TPM1) account for 1–5%. Non-sarcomeric gene variants, including ACTN2, ALPK3, and CSRP3, are rarer but relevant to its etiology. These discoveries have facilitated precision medicine approaches [6,7]. In line with these findings, next-generation sequencing (NGS) and expanded gene panels have improved diagnostic precision and differentiation of HCM from phenocopies like Fabry disease (GLA) and transthyretin cardiac amyloidosis (TTR). Despite overlapping phenotypic features, these conditions have distinct etiologies, prognoses, and treatments, underscoring the importance of accurate differential diagnosis [8].

From a clinical standpoint, HCM is highly variable, ranging from asymptomatic cases to those with dyspnea, chest pain, or syncope. Although it can manifest at any age, it is often diagnosed in adolescence or early adulthood. Disease progression is associated with complications such as arrhythmias and SCD [5,9]. Management options include pharmacological therapy, septal myectomy, alcohol septal ablation, and implantable cardioverter defibrillators (ICDs), which improve quality of life and reduce the risk of heart failure and SCD [10].

In addition, recent studies highlight the clinical and genetic differences in HCM between ethnic groups. In Brazil, increased mortality rates have been reported in the Northeast and Southeast regions, predominantly affecting white men over 40 years age. Researchers advocate for early diagnosis protocols and effective therapeutic strategies to reduce mortality and improve quality of life [11]. Consequently, genetic testing plays a crucial role in etiological diagnosis, family screening, and distinguishing HCM from other causes of LVH. The CLINGEN consortium has identified eight genes with definitive associations with HCM, highlighting the importance of these tests in clinical management and emerging therapies, such as gene-based treatments. For relatives of individuals with pathogenic variants, predictive tests and regular clinical surveillance, including ECG and echocardiography, are recommended according to current guidelines [12,13].

In this context, the objective of this study is to analyze the genetic, clinical, and epidemiological profile of patients with a previous diagnosis of hypertrophic cardiomyopathy (positive phenotype) and asymptomatic individuals with a family history of sudden death and/or unconfirmed HCM.

## 2. Materials and Methods

### 2.1. Study Design

This prospective, cross-sectional study was conducted between June 2021 and August 2024. This study involved non-random, consecutive samples of patients with a previous diagnosis of HCM, referred by cardiologists. This research adhered to ethical standards and was approved by the Research Ethics Committee at the Federal University of Sergipe (CAAE 50634021.0.0000.5546, opinion: 5.793.007).

### 2.2. Inclusion Criteria

Adults aged 18 years or older with a confirmed diagnosis of HCM based on echocardiography and/or cardiac magnetic resonance imaging (CMR), following the diagnostic criteria established by the American Heart Association (AHA), a family history of HCM, or a family history of unconfirmed HCM and/or sudden cardiac death (SCD were included).

### 2.3. Exclusion Criteria

Individuals with hypertrophy attributable to other known causes such as coronary artery disease, valvopathies, hypertensive cardiomyopathy, or dilated cardiomyopathy were excluded.

### 2.4. HCM Diagnosis Definition

The diagnosis of HCM was established by transthoracic echocardiography (TTE) based on specific criteria, such as maximum ventricular hypertrophy (LVH) unexplained by other causes ≥15 mm in any segment of the left ventricle or ≥13 mm in individuals with a family history of HCM. Obstructive presentations were defined by a gradient ≥ 30 mmHg in the left ventricular outflow tract, at rest, after Valsalva maneuver, or in orthostasis.

### 2.5. Molecular Testing

Genetic confirmation of HCM diagnosis was defined as the primary outcome, i.e., positive phenotype/genotype. Individuals with pathogenic or likely pathogenic variants in the genes tested were classified as having a positive genotype. Genes analyzed included ACTC1, FLNC, LAMP2, MYL2, PRKAG2, TNNI3, TTR, CSRP3, GLA, MYBPC3, MYL3, PTPN11, TNNT2, DES, JPH2, MYH7, PLN, TNNC1, and TPM.

### 2.6. Sample Characterization Variables

Sociodemographic variables included gender, age, origin, income, and type of healthcare assistance. Clinical variables included comorbidities such as diabetes, hypertension, and dyslipidemia. Additionally, this study recorded data on hypertrophic ventricular wall thickness (in millimeters) and clinical manifestations, including acroparesthesias, angiokeratomas, anhidrosis, arrhythmias, psychiatric disorders (anxiety and depression). Other clinical signs, such as elevated serum creatinine, corneal verticillata, hypoacusis, heart failure, renal insufficiency (microalbuminuria, proteinuria), and gastrointestinal disorders, were also noted.

### 2.7. Statistical Analysis

Statistical analysis was performed using R software (Version 2024.09.0, Build 375) [14]. Categorical variables are expressed as absolute numbers and percentages, with 95% confidence intervals. The chi-squared or Fisher’s exact test was applied for categorical variables. Continuous variables are expressed as mean ± standard error, and distributions were analyzed with the Kolmogorov–Smirnov test. For non-parametric distributions, the Mann–Whitney U-test was used to compare groups.

### 2.8. Ethical and Legal Principles

This study followed the ethical principles of the Declaration of Helsinki and was approved by the Research Ethics Committee at the Federal University of Sergipe. Participation was voluntary, with all participants being informed about the risks and benefits of this study. Participants provided written informed consent (Termo de Consentimento Livre e Esclarecido—TCLE), ensuring compliance with ethical and legal requirements.

## 3. Results

In this study, 200 patients from 152 distinct families were analyzed. Among them, 130 individuals (65% [95% C.I.: 57.9–71.6]) fully met the diagnostic criteria for hypertrophic cardiomyopathy (HCM) established by the American Heart Association (AHA). The remaining 70 participants had a family history of unconfirmed HCM or sudden cardiac death and underwent genetic testing. The sample consisted of 115 males (58%), with a mean age of 53 years (standard deviation: 17.0). Of the participants, 77 (39%) were dependent on the Brazilian public healthcare system.

The most prevalent comorbidities were systemic arterial hypertension (61%), diabetes mellitus (20%), and dyslipidemia (58%). Additionally, 5.1% of the patients had an implantable cardioverter defibrillator, and 28% were classified as obese to varying degrees. Regarding pharmacological treatment, the most frequently prescribed medications included beta-blockers (68%), statins (59%), and angiotensin-converting enzyme inhibitors/angiotensin receptor blockers (ACEIs/ARBs), prescribed to 52% of the participants.

### 3.1. Genetic Analysis

**Figure 1:**
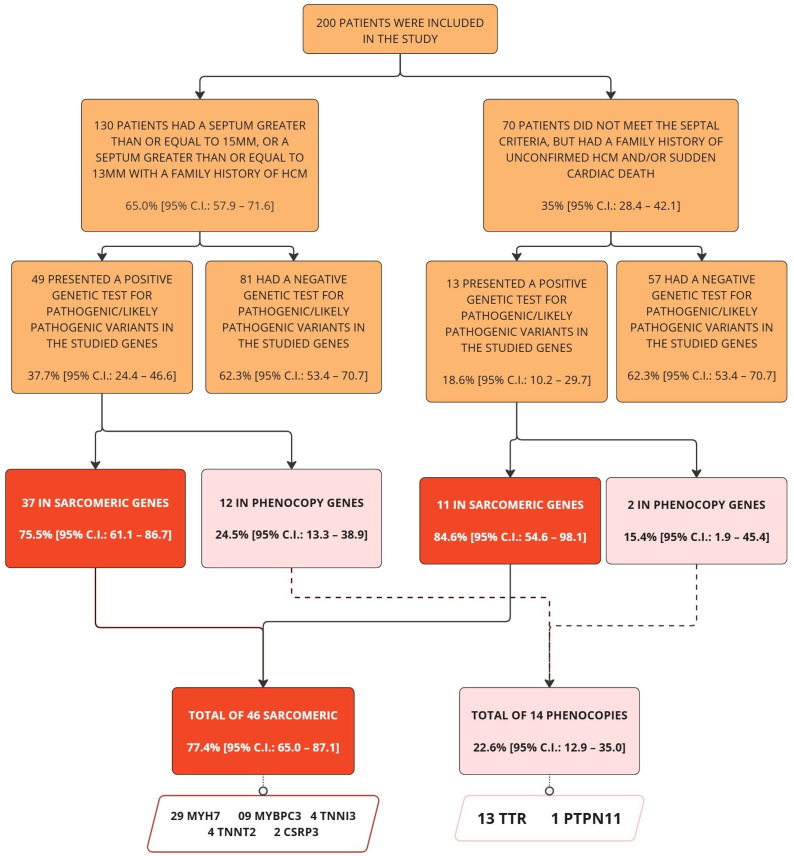
**Flowchart of criteria and genetic diagnosis.** The flowchart depicts the subdivision created within the sample and highlights the genetic diagnoses across subgroups and the total sample.

The criteria for patient inclusion and the genetic diagnostic workflow are summarized in Figure 1. Among the sample, 62 patients (31% [95% C.I.: 24.7–37.9]) tested positive for variants classified as pathogenic or likely pathogenic (P/LP) through genetic analysis, whereas 138 (69% [95% C.I.: 62.1–75.3]) were genotype-negative. Within the genotype-positive subgroup, 77.4% (95% C.I.: 65.0–87.1) exhibited genetic alterations in sarcomeric genes, while 22.6% (95% C.I.: 12.9–35.0) harbored variants in genes associated with phenocopies. Among genotype-negative patients, 89 distinct variants of uncertain significance (VUSs) were identified in 81 individuals, accounting for 58.7% (95% C.I.: 50.0–67.0) of this group.

**Figure 2:**
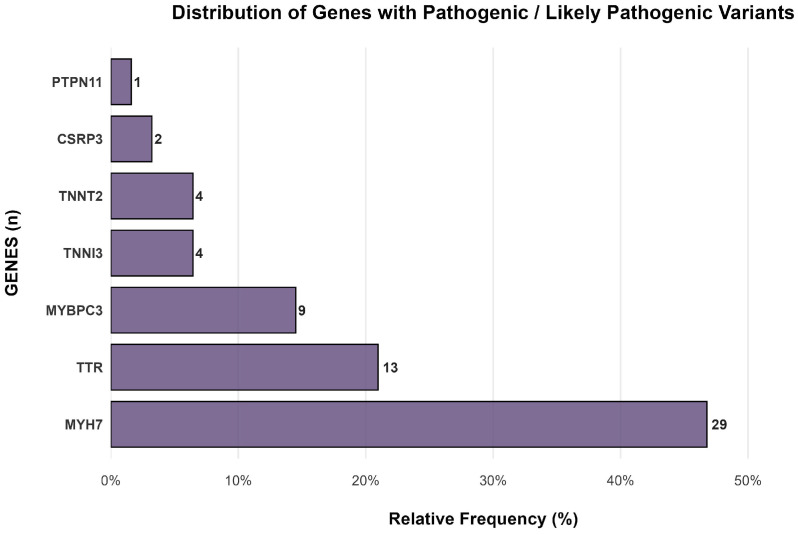
**Distribution of genes with pathogenic/likely pathogenic variants in genetically positive patients.** This horizontal bar plot shows the relative frequency of genes carrying pathogenic (P) or likely pathogenic (LP) variants among patients with a positive genetic diagnosis. The length of each bar represents the proportion of patients carrying a variant in that specific gene. Absolute counts are shown on the bars, and the bars are ordered from most to least frequent variants for better visualization.

The most frequent genes in our study were MYH7, observed in 29 out of 62 cases (46.77% [95% C.I.: 34.35–59.19%]), TTR in 13 cases (20.97% [95% C.I.: 10.83–31.10%]), and MYBPC3 in 9 cases (14.52% [95% C.I.: 5.75–23.28%]). Figure 2 shows the distribution of genes with pathogenic or likely pathogenic variants in genetically positive patients. A list of all genes is available in Table A1 in Appendix A.

When stratified by gene, the subgroup with pathogenic/likely pathogenic variants in the TTR gene had a median age of 77 years (IQR: 66–79), significantly older than the subgroup with sarcomeric gene variants, which had a median age of 45 years (IQR: 35–59, *p* < 0.001).

**Figure 3:**
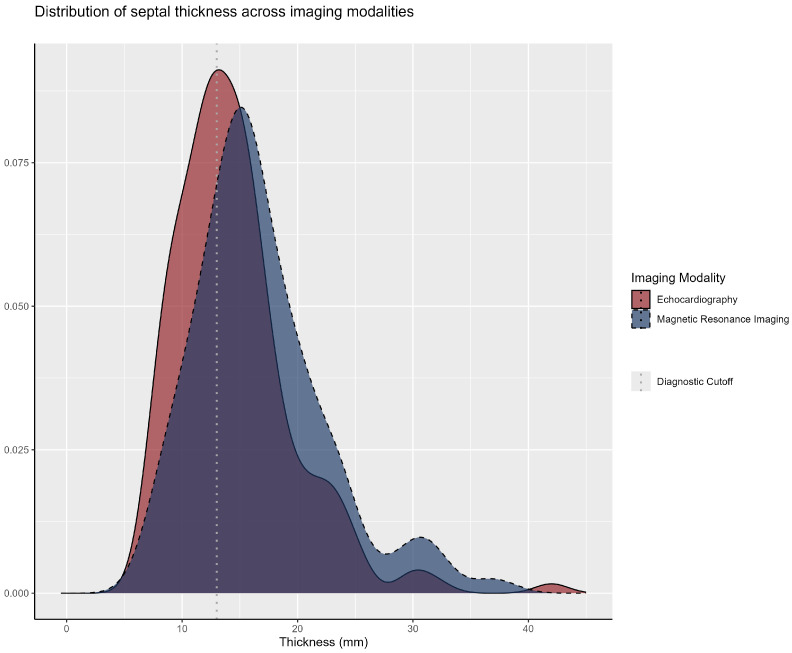
**Septal thickness distribution across imaging modalities (echocardiography vs. cardiac MRI).** The distribution of interventricular septal thickness (measured in millimeters) according to imaging technique: echocardiography (solid red line) and cardiac MRI (dashed bluenline). The vertical line at 13 mm (dark gray dotted line) represents the diagnostic cutoff for hypertrophic cardiomyopathy (HCM) as defined by the 2024 AHA/ACC/AMSSM/HRS/PACES/SCMR guideline.

The median interventricular septal thickness was 14.1 mm (IQR: 11.0–17.0) on echocardiography and 16.0 mm (IQR: 13.0–20.0) on cardiac magnetic resonance imaging, with an overall median of 15.0 mm (IQR: 13.0–18.0) when considering either diagnostic modality. The analysis of these diagnostic approaches demonstrated their comparable results, as illustrated in Figure 3.

**Figure 4:**
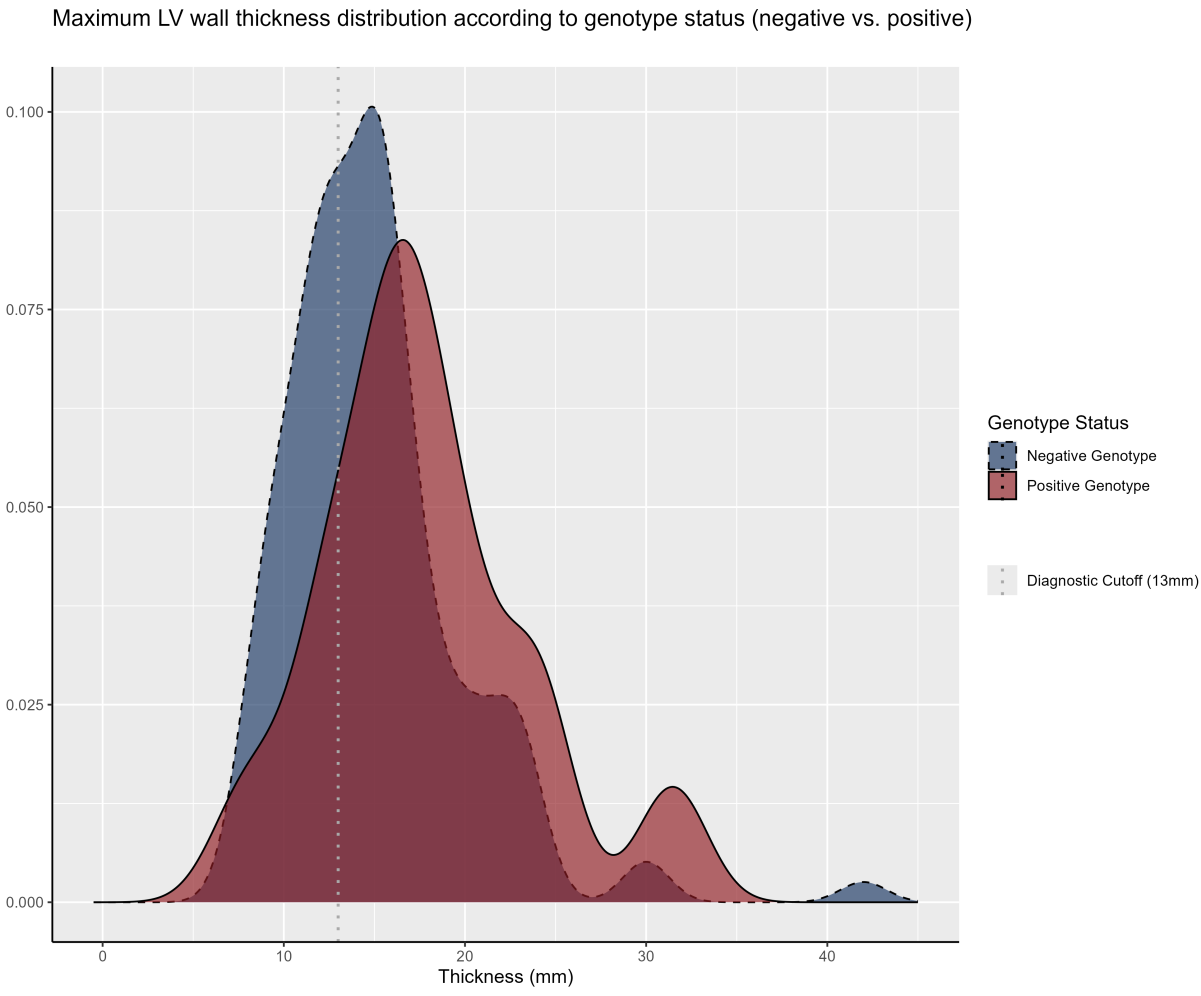
**Maximum LV wall thickness distribution according to genotype status (negative vs. positive).** The distribution of left ventricular wall thickness (measured in millimeters) for genetic diagnosis subgroups: positive genotype (solid red line) and negative genotype (dashed blue line). The dotted vertical line at 13 mm represents the minimal diagnostic cutoff for HCM based on the 2024 AHA/ACC/AMSSM/HRS/PACES/SCMR guideline.

When stratified by genetic diagnosis, patients with a positive genotype exhibited a greater mean interventricular septal thickness (17.7 mm vs. 15.0 mm; *p* < 0.001). The median septal thickness among patients with VUSs and those without pathogenic/likely pathogenic variants was 14.4 mm and 16.0 mm, respectively. Figure 4 illustrates the distribution of septal thickness across genetic diagnosis subgroups.

### 3.2. Clinical Manifestations and Family History

Regarding clinical signs and symptoms, 61% of the sample reported palpitations, with a higher prevalence in the genotype positive group (71% vs 59%; *p* = 0.042). Dyspnea was observed in 45% of the total sample, with no significant differences between the groups (*p* = 0.5). Syncope occurred in 27% of patients, with similar proportions across groups (*p* = 0.7). Precordial pain was reported by 44% of the participants, again without significant differences between the groups (*p* = 0.5). Specific manifestations, including angiokeratoma, hypoacusis, and acroparesthesia, also showed no significant variation between groups.

Family history played a pivotal role in characterizing the sample. Parental consanguinity was observed in 11% of cases, while only one individual (0.5%) reported a family history of Fabry disease. The recurrence of sudden cardiac death among first- and/or second-degree relatives was reported by 53% of patients diagnosed with HCM, with a higher prevalence in the genotype-positive group (68% vs. 46%; *p* = 0.004), as illustrated in Table 1.

## 4. Discussion

This study evaluated 200 patients through genetic testing for variants in 19 genes associated with hypertrophic cardiomyopathy (HCM) and its phenocopies. The analysis revealed a positivity rate of 31% for pathogenic or likely pathogenic variants, with 24% identified in sarcomeric genes and 7% in genes related to phenocopies. These findings align with the medical literature, which reports a diagnostic yield of genetic testing for HCM ranging between 30% and 60% [13,15,16]. Moreover, a recent multicenter, international study by Silva et al. (2024), utilizing a similar genetic panel, reported a slightly lower positivity rate of 24.7%, with 21.5% attributed to sarcomeric gene variants and 3.2% to phenocopies [17]. The median age at HCM diagnosis in our cohort was 48 years. Evidence from meta-analyses and international registries has shown similar ages at presentation while also highlighting variations depending on the underlying gene and family context [18,19].

In terms of gene prevalence, the literature commonly cites MYBPC3 as the most frequent sarcomeric gene, found in 40–45% of cases, followed by MYH7, present in 15–25% of cases. In contrast, our study identified MYH7 and MYBPC3 variants in 46.77% and 14.5% of cases, respectively [3,5,20]. An additional noteworthy observation was the high frequency (6.5%) of variants in the TTR gene within our cohort. While data on the prevalence of TTR variants in the HCM population remain limited, previous studies have reported frequencies ranging from 0.7% to 1.2%. Thus, this higher prevalence in our sample may reflect regional genetic variability or a potential confounder effect [21,22].

Regarding variants of uncertain significance (VUSs), our study identified 89 such variants in 81 patients. The medical literature suggests that the penetrance of variants associated with HCM is variable and may be influenced by genetic and environmental factors that are not yet fully understood. Consequently, monitoring these patients, classified in this study as genotype-negative, is crucial due to the possibility of phenotypic conversion over time. This enables the early identification of a transition to a clinical HCM phenotype, allowing for timely therapeutic intervention [3,18].

Another important finding was that 92.3% of patients, despite originating from different families, with pathogenic or likely pathogenic (P/LP) variants in the TTR gene carried the same variant, Val142Ile, primarily associated with the cardiac phenotype of amyloidosis. Furthermore, these patients were older on average than those with sarcomeric gene variants [23]. According to Reddi, the V122I mutation is associated with late-onset disease, frequently observed in individuals of African descent, typically manifesting during the seventh decade of life. In line with this, Reddi’s study reported a mean disease onset age of approximately 72 years for heterozygotes, whereas in our study, the mean onset age was 70 years [24].

From a structural perspective, we observed a higher median maximum left ventricular wall thickness (MLVWT) in the genotype-positive subgroup compared to the genotype-negative subgroup (17.7 mm vs. 15.0 mm; *p* < 0.001). The literature suggests that patients with mutations in sarcomeric genes tend to exhibit greater interventricular wall thickness, often in an asymmetric pattern, with a preference for the apical and anteroseptal regions, and are at increased risk for adverse cardiac events throughout their lifetime. Notably, no significant differences were observed between groups or within individual patients in left ventricular measurements obtained through cardiac magnetic resonance imaging (CMR) and echocardiography. This consistency across imaging modalities supports the reliability of these methods for characterizing cardiac morphology in patients with HCM [25,26].

In terms of clinical outcomes, our study also observed that patients with P/LP variants in sarcomeric genes exhibited a higher incidence of sudden cardiac death (SCD) among first- and/or second-degree relatives. The presence of mutations in sarcomeric genes is associated with an increased risk of SCD [27]. A study using the Sarcomeric Human Cardiomyopathy Registry (SHaRe) reported that the incidence of SCD was approximately twice as high in genotype-positive individuals compared to genotype-negative patients [28].

Carriers of pathogenic or likely pathogenic (P/LP) variants in MYH7 generally present with an earlier onset of hypertrophic cardiomyopathy, more pronounced left ventricular hypertrophy, and an increased incidence of adverse outcomes, including ventricular arrhythmias and sudden cardiac death, particularly in individuals with a family history of early cardiac events. These findings reflect the high penetrance and early phenotypic expression associated with MYH7 mutations [3,29]. In contrast, mutations in MYBPC3, the most prevalent genetic cause of hypertrophic cardiomyopathy, exhibit considerable clinical heterogeneity, ranging from asymptomatic carriers to patients with advanced heart failure and sudden cardiac death. Long-term follow-up data indicate that MYBPC3 carriers are more prone to progressive systolic dysfunction compared with those harboring MYH7 variants [30,31]. Additionally, less frequent pathogenic variants in other sarcomeric genes, including TNNI3, TNNT2, and TPM1, remain clinically significant due to their association with arrhythmic risk and potential responsiveness to septal reduction therapies in obstructive forms of the disease [3,32].

Genetic panels are a relatively recent advancement in medical history and remain largely inaccessible in emerging countries. Consequently, there is a significant portion of the population that may harbor pathogenic or likely pathogenic (P/LP) variants but remain underdiagnosed due to the proband’s premature death, often from sudden cardiac events. Within this framework, this study highlights a higher prevalence of these variants in the examined cohort compared to global prevalence rates, reinforcing the importance of the 2024 AHA/ACC/AMSSM/HRS/PACES/SCMR guideline when advocating for genetic testing in cases with specific clinical indications or under the guidance of a cardiovascular genetics specialist, underscoring its value in improving diagnosis, risk stratification, and familial screening efforts [3,6,11].

Turning to clinical manifestations, palpitations were more prevalent in the genotype-positive group compared to the genotype-negative group. A systematic review and meta-analysis demonstrated that mutations in sarcomeric genes are associated with an elevated risk of ventricular tachycardia, syncope, and heart failure. Additionally, the greater hypertrophy observed in this group may contribute to a higher prevalence of associated symptoms [27,33].

With respect to comorbidities, analysis of body mass index (BMI) and related conditions revealed that 28% of patients were classified as obese, 61% had hypertension, 58% had dyslipidemia, and 20% had diabetes mellitus (DM). The literature highlights hypertension as a common comorbidity in patients with HCM, with a prevalence ranging from 35% to 50% in adults. In a Russian cohort of 193 patients, 63% presented with hypertension, 31% with obesity, and 11% with diabetes, showing that cardiovascular comorbidities are consistently prevalent among individuals with HCM across populations [34]. Moreover, studies suggest that patients with coexisting HCM and DM exhibit a higher prevalence of diastolic dysfunction, pulmonary hypertension, and significant mitral regurgitation [35]. In addition, dyslipidemia has been linked to an increased incidence of HCM, particularly in younger individuals, suggesting that dyslipidemia may influence the clinical expression of HCM. Therefore, the effective management of these comorbidities is critical to optimizing clinical outcomes and improving the overall prognosis of patients with HCM [3,36].

Nevertheless, this study has limitations that should be considered. The use of a consecutive, single-region sample may restrict generalizability and under-represent asymptomatic carriers or those with limited access to care. Its cross-sectional design precludes evaluation of disease progression, long-term genotype–phenotype correlations, and future reclassification of variants of uncertain significance. Furthermore, the 19-gene panel excluded recently described genes and non-coding regions, possibly leading to underdiagnosis.

Building on these limitations, future investigations involving larger, multicenter, and ethnically diverse cohorts, incorporating whole-exome or whole-genome sequencing, longitudinal follow-up, and advanced imaging modalities such as cardiac MRI, will be crucial for refining risk stratification, guiding personalized therapeutic strategies, and elucidating the impact of genetic background, environmental factors, and comorbidities on disease progression. Such approaches may clarify the penetrance and expressivity of both rare and common variants, identify population-specific founder mutations, assess long-term clinical outcomes, and inform the development of tailored interventions for distinct patient subgroups.

## 5. Conclusions

Looking ahead, the future of diagnosing and managing HCM is promising, especially with continued advancements in genetic testing and imaging technologies such as cardiac MRI. In particular, this study revealed a higher prevalence of TTR-related cardiac amyloidosis, indicating that regional factors might play a role in genetic variations. Accordingly, family history and clinical symptoms will become even more important in guiding when genetic testing is needed, leading to earlier diagnoses and more precise risk assessments. As a result, with greater access to genetic tests and more personalized treatment options, we can expect significant improvements in care and outcomes for patients with HCM and its related conditions.

In line with these perspectives, the frequency of pathogenic variants in sarcomeric genes aligns with the existing literature, but TTR-related cardiac amyloidosis was notably higher, suggesting greater prevalence in this population. Moreover, interventricular septal thickness >17 mm and a family history of SCD were strong predictors of positive genetic tests. Therefore, while genetic testing should be guided by family screening and counseling, a family history of SCD or unconfirmed HCM may justify testing, especially when a pathogenic variant is identified in a family member.

## Figures and Tables

**Table 1:** Clinical and demographic characteristics of the study population by genetic diagnosis.

	Overall (*N* = 200)	Positive Genotype (*n* = 62)	Negative Genotype (*n* = 138)	*p*-Value ^1^
**Sample Profile**				
Median Age, (median [IQR])	52 (41–66)	48 (38–67)	54 (43–65)	0.2
Men, n (%)	115 (58%)	30 (48%)	85 (62%)	0.081
Body Mass Index, kg/m^2^ (median [IQR])	27.4 (25.2–30.5)	27.4 (25.5–30.7)	27.3 (24.2–30.0)	0.5
**Signs and Symptoms, n (%)**				
Palpitations	121 (61%)	44 (71%)	77 (56%)	**0.042**
Cornea Verticillata	3 (1.5%)	2 (3.3%)	1 (0.7%)	0.2
Angiokeratomas	11 (5.5%)	5 (8.1%)	6 (4.3%)	0.3
Dyspnea	89 (45%)	30 (48%)	59 (43%)	0.5
Syncope	52 (27%)	17 (28%)	35 (26%)	0.7
Precordial Pain	87 (44%)	25 (40%)	62 (46%)	0.5
Acroparesthesias	74 (37%)	25 (40%)	49 (36%)	0.5
**Comorbidity, n (%)**				
Hypertension	120 (61%)	34 (55%)	86 (63%)	0.3
Diabetes	40 (20%)	8 (13%)	32 (23%)	0.093
Dyslipidemia	114 (58%)	32 (52%)	82 (60%)	0.3
ICD Carrier	9 (5.1%)	6 (11%)	3 (2.5%)	**0.026**
Hypothyroidism	17 (9.0%)	8 (15%)	9 (6.7%)	0.10
Heart Failure	52 (27%)	18 (31%)	34 (26%)	0.5
Renal Failure	12 (6.6%)	3 (5.2%)	9 (7.3%)	0.8
**Medications, n (%)**				
Beta-Blockers	115 (68%)	42 (78%)	73 (63%)	0.054
ACE Inhibitors/ARBs	88 (52%)	19 (37%)	69 (58%)	**0.011**
Statins	101 (59%)	27 (52%)	74 (63%)	0.2
Amiodarone	17 (11%)	9 (20%)	8 (7.3%)	**0.026**
Diuretics	67 (41%)	20 (41%)	47 (41%)	>0.9
Aspirin (ASA)	35 (22%)	7 (15%)	28 (25%)	0.15
**Habits, n (%)**				
Physical Inactivity	71 (38%)	23 (38%)	48 (38%)	>0.9
Smoking	29 (16%)	6 (10%)	23 (18%)	0.2
Alcohol Consumption	35 (19%)	10 (17%)	25 (20%)	0.7
**Family History, n (%)**				
Parental Consanguinity	22 (11%)	4 (6.7%)	18 (14%)	0.13
Sudden Cardiac Death	101 (53%)	41 (68%)	60 (46%)	**0.004**
**Image-Derived Measurements, n (IQR)**				
LV Wall Thickness	15.0 (12.0–18.0)	17.0 (14.2–20.6)	15.0 (12.0–17.0)	**<0.001**
Ejection Fraction ^2^	66 (61–71)	67 (62–72)	65 (61–70)	0.2
Left Ventricular Diastolic Diameter ^2^	48 (43–53)	46 (42–52)	48 (44–55)	**0.030**
Left Atrial Diameter ^2^	42 (37–48)	45 (39–49)	41 (36–46)	**0.029**

^1^ Wilcoxon rank sum test; Pearson’s chi-squared test; Fisher’s exact test; ^2^ echocardiography.

## Data Availability

The data supporting the findings of this study are publicly available from the project’s GitHub repository at https://github.com/bielkuhn/HCM_Article_UFS (30 August 2025). This repository contains the source code used for data analysis and visualization, the input and output data files utilized in the analyses, and documentation describing the data, methodology, and instructions to reproduce the results.

## Abbreviations

The following abbreviations are used in this manuscript:

ACC	American College of Cardiology
ACEI	Angiotensin-Converting Enzyme Inhibitor
AHA	American Heart Association
AMSSM	American Medical Society for Sports Medicine
ARB	Angiotensin Receptor Blocker
BMI	Body Mass Index
DM	Diabetes Mellitus
ESC	European Society of Cardiology
GLA	Gene Associated with Fabry Disease
HCM	Hypertrophic Cardiomyopathy
HRS	Heart Rhythm Society
ICD	Implantable Cardioverter Defibrillator
IQR	Interquartile Range
LVH	Left Ventricular Hypertrophy
MLVWT	Maximum Left Ventricular Wall Thickness
MRI	Magnetic Resonance Imaging
MYBPC3	Myosin-Binding Protein C3
MYH7	Myosin Heavy Chain 7
NGS	Next-Generation Sequencing
P/LP	Pathogenic or Likely Pathogenic
PACES	Pediatric and Congenital Electrophysiology Society
RMC	Cardiac Magnetic Resonance
SCD	Sudden Cardiac Death
SCMR	Society for Cardiovascular Magnetic Resonance
TCLE	Termo de Consentimento Livre e Esclarecido (Informed Consent Form)
TTE	Transthoracic Echocardiography
TTR	Transthyretin
VUS	Variant of Uncertain Significance

## Appendix A

**Table A1:** Genetic variants identified in the study population. The table lists the genetic variants identified in this study; each row details the affected gene, ACMG classification, nucleotide change, and amino acid change.

Family	Gene	Variant	Amino Acid Change	ACMG Classification
CX	*CSRP3*	536C>T	Thr179Met	Likely Pathogenic
XX	*CSRP3*	715G>A	Asp239Asn	Pathogenic
CXIX	*MYBPC3*	3662del	Leu1221Argfs*16	Pathogenic
CXXX	*MYBPC3*	3662del	Leu1221Argfs*16	Pathogenic
CXXX	*MYBPC3*	3662del	Leu1221Argfs*16	Pathogenic
I	*MYBPC3*	1484G>A	Arg495Gln	Pathogenic
I	*MYBPC3*	1484G>A	Arg495Gln	Pathogenic
LI	*MYBPC3*	1800del	Lys600Asnfs*2	Pathogenic
LI	*MYBPC3*	1800del	Lys600Asnfs*2	Likely Pathogenic
XLV	*MYBPC3*	Arg453Cys	Leu1221Argfs*16	Pathogenic
XXVIII	*MYBPC3*	3662del	Leu1221Argfs*16	Pathogenic
CIII	*MYH7*	2389G>A	Ala797Thr	Pathogenic
CIII	*MYH7*	2389G>A	Ala797Thr	Pathogenic
CXII	*MYH7*	1357C>T	Arg453Cys	Pathogenic
CXX	*MYH7*	715G>A	Asp239Asn	Pathogenic
CXXI	*MYH7*	1357C>T	Arg453Cys	Pathogenic
CXXXI	*MYH7*	2167C>G	Arg723Gly	Pathogenic
LIX	*MYH7*	1357C>T	Arg453Cys	Pathogenic
LV	*MYH7*	715G>A	Asp239Asn	Pathogenic
LVII	*MYH7*	715G>A	Asp239Asn	Pathogenic
LVII	*MYH7*	1750G>C	Gly584Arg	Pathogenic
LVIII	*MYH7*	715G>A;p	Asp239Asn	Pathogenic
LXI	*MYH7*	746G>A	Arg249Gln	Pathogenic
LXXV	*MYH7*	2389G>A	Ala797Thr	Likely Pathogenic
LXXV	*MYH7*	2389G>A	Ala797Thr	Likely Pathogenic
LXXV	*MYH7*	2389G>A	Ala797Thr	Pathogenic
LXXXIV	*MYH7*	2389G>A	Ala797Thr	Likely Pathogenic
XCIX	*MYH7*	2012G>A	Arg671His	Pathogenic
XI	*MYH7*	1357C>T	Arg453Cys	Pathogenic
XII	*MYH7*	1357C>T	Arg453Cys	Pathogenic
XIX	*MYH7*	1750G>C	Gly584Arg	Pathogenic
XLIX	*MYH7*	2389G>A	Ala797Thr	Pathogenic
XLVI	*MYH7*	1750G>C	Gly584Arg	Pathogenic
XVII	*MYH7*	1750G>C	Gly584Arg	Pathogenic
XXXV	*MYH7*	715G>A	Asp239Asn	Pathogenic
XXXVII	*MYH7*	2389G>A	Ala797Thr	Pathogenic
III	*PTPN11*	836A>G	Tyr279Cys	Pathogenic
CIX	*TNNI3*	575G>A	Arg192His	Pathogenic
XVIII	*TNNI3*	470C>T	Ala157Val	Pathogenic
CXLI	*TNNT2*	856C>T	Arg286Cys	Pathogenic
CXXVIII	*TNNT2*	418C>T	Arg140Cys	Pathogenic
CXXXIX	*TNNT2*	877C>T	Arg293Cys	Pathogenic
LII	*TNNT2*	877C>T	Arg293Cys	Likely Pathogenic
CLI	*TTR*	424G>A	Val142Ile	Pathogenic
CXXVII	*TTR*	424G>A	Val142Ile	Pathogenic
L	*TTR*	424G>A	Val142Ile	Pathogenic
LVI	*TTR*	424G>A	Val142Ile	Pathogenic
LXIII	*TTR*	424G>A	Val142Ile	Pathogenic
LXXIX	*TTR*	424G>A	Val142Ile	Pathogenic
LXXXIII	*TTR*	424G>A	Val142Ile	Pathogenic
XCII	*TTR*	424G>A	Val142Ile	Pathogenic
XIV	*TTR*	424G>A	Val142Ile	Pathogenic
XLVIII	*TTR*	424G>A	Val142Ile	Pathogenic
XXV	*TTR*	424G>A	Val142Ile	Pathogenic
XXV	*TTR*	5330-2A>G	Splice acceptor	Likely Pathogenic
